# Composition and diversity of soil microbial communities change by introducing *Phallus impudicus* into a *Gastrodia elata* Bl.-based soil

**DOI:** 10.1186/s12866-024-03330-4

**Published:** 2024-06-08

**Authors:** Yanhong Wang, Jiao Xu, Qingsong Yuan, Lanping Guo, Gang Zheng, Chenghong Xiao, Changgui Yang, Weike Jiang, Tao Zhou

**Affiliations:** 1 Resource Institute for Chinese and Ethnic Materia Medica, Guizhou University of Traditional Chinese Medicine, Guiyang, China; 2 State Key Laboratory for Quality Ensurance and Sustainable Use of Dao-di Herbs, National Resource Center for Chinese Materia Medica, China Academy of Chinese Medical Sciences, Beijing, China; 3 The Second Affiliated Hospital of Zunyi Medical University, Zunyi, China

**Keywords:** Traditional chinese medicine, Soil nutrient, High-throughput sequencing, *Armillaria gallica*

## Abstract

**Background:**

The *Gastrodia elata* Bl. is an orchid, and its growth demands the presence of *Armillaria* species. The strong competitiveness of *Armillaria* species has always been a concern of major threat to other soil organisms, thus disrupting the equilibrium of soil biodiversity. Introducing other species to where *G. elata* was cultivated, could possibly alleviate the problems associated with the disequilibrium of soil microenvironment; however, their impacts on the soil microbial communities and the underlying mechanisms remain unclear. To reveal the changes of microbial groups associated with soil chemical properties responding to different cultivation species, the chemical property measurements coupled with the next-generation pyrosequencing analyses were applied with soil samples collected from fallow land, cultivation of *G. elata* and *Phallus impudicus*, respectively.

**Results:**

The cultivation of *G. elata* induced significant increases (*p* < 0.05) in soil pH and NO_3_-N content compared with fallow land, whereas subsequent cultivation of *P. impudicus* reversed these *G. elata-*induced increases and was also found to significantly increase (*p* < 0.05) the content of soil NH_4_^+^-N and AP. The alpha diversities of soil microbial communities were significantly increased (*p* < 0.01) by cultivation of *G. elata* and *P. impudicus* as indicated with Chao1 estimator and Shannon index. The structure and composition of soil microbial communities differed responding to different cultivation species. In particular, the relative abundances of *Bacillus*, *norank_o_Gaiellales*, *Mortierella* and *unclassified_k_Fungi* were significantly increased (*p* < 0.05), while the abundances of potentially beneficial genera such as *Acidibacter*, *Acidothermus*, *Cryptococcus*, and *Penicillium* etc., were significantly decreased (*p* < 0.05) by cultivation of *G. elata*. It’s interesting to find that cultivation of *P. impudicus* increased the abundances of these genera that *G. elata* decreased before, which contributed to the difference of composition and structure. The results of CCA and heatmap indicated that the changes of soil microbial communities had strong correlations with soil nutrients. Specifically, among 28 genera presented, 50% and 42.9% demonstrated significant correlations with soil pH and NO_3_-N in response to cultivation of *G. elata* and *P. impudicus*.

**Conclusions:**

Our findings suggested that the cultivation of *P*. *impudicus* might have potential benefits as result of affecting soil microorganisms coupled with changes in soil nutrient profile.

**Supplementary Information:**

The online version contains supplementary material available at 10.1186/s12866-024-03330-4.

## Background

More than 80% extant land plant species establish associations with mycorrhizal fungi to exchange key nutrients [1]. Orchidaceae showing mycorrhizal association with fungi, are partially or fully dependent on them during their life-cycle. The rare herb, *Gastrodia elata* Bl., a member of the orchid family, is widespread in Asian countries and valued as both medicine and food [2]. Evidence showed that the *G. elata* has abandoned photosynthetic genes and lives in symbiotic association with *Mycena* spp. and *Armillaria* species, e.g. *Armillaria gallica*, or *Armillaria mellea*, from germination to maturity to obtain its required nutrition [3].

In China, *G. elata* has been used for over 2,000 years in the treatment of headache, vertigo, and hemiplegia [4, 5]. For a long time, wild populations were sufficient to meet the needs of individuals suffering from such diseases. However, overexploitation has resulted in a significant decline in wild populations [6, 7]. Considerable efforts in artificial cultivation of *G. elata* were made to meet the growing demand, and the domestication of the wild *G. elata* plant succeeded until the late 1960s [8].

Modern cultivation practices of *G. elata* are often conducted under monoculture, which usually fails due to replanting issues. Successful re-cultivation requires leaving the land uncultivated for more than 3 years. Replanting activities have caused major supply bottlenecks not only for *G. elata* but also many other medicinal herbs, such as *Pinellia ternate* [9] and American ginseng [10]. These issues are often associated with allelopathy, autotoxicity or changes in soil physicochemical properties and soil microflora [9–11]. It is commonly believed that the replanting activities of *G. elata* are related to its essential symbiotic *Armillaria* species [12]. Because the *Armillaria* members usually compete with other microbes associated with secondary metabolites [13, 14], which would cause the simplification or disequilibrium of the biodiversity in soil environment.

There are no doubts that soil microenvironment comprises of abundant and complex soil microorganisms [15]. Their diversity and composition play significant roles in maintaining soil functions through involvement in the turnover of soil nutrients [16, 17]. Soil micro-ecological imbalance is considered as one of the critical factors associated with replanting work [18]. Evidence indicated that 3 years of continuous cultivation of American ginseng decreased the soil bacterial diversity, while increasing the fungal diversity [10]. Meanwhile, others revealed that soil microbial diversity and structure can gain benefits from the rotation of wheat with *Pinellia ternate* [9]. Therefore, if crop rotation can recover the stability of the microbiome which is beneficial to agricultural production, rotation management could be an environmental-friendly, sustainable, and low-cost remediation technology with a high efficiency [19]. The key point is to identify and distinguish the effects of rotation management on soil micro-ecological equilibrium.

To better utilize the land resources and maintain the ecology of the forest environment, farmers use an edible fungus, namely *Phallus impudicus* to cultivate after *G. elata*. A previous study showed that *P*. *impudicus* is capable of decomposing substrates and have been evidenced to be able to allocate, uptake and store nutrients [20]. However, how the cultivation of *P*. *impudicus* will influence the soil microenvironment remains unknown. Studies focusing on the equilibrium of soil microorganisms and soil biochemical properties in cultivation of *G. elata* and *P*. *impudicus* are needed. Herein, this study provided an insight into the soil biochemical properties and soil microbial communities (including both bacterial and fungal communities) associated with cultivation of *G. elata* and *P*. *impudicus*. The soil microenvironment before and after cultivation of *G. elata* and *P*. *impudicus* would also be disclosed. In so doing, it is intended to understand the effect of different cultivation practices of saprotrophic fungi on the soil microenvironment, and to elucidate the efficacy of this strategy (Fig. S1).

## Materials and methods

### Description of the trial site and experimental design

The trial site is located at Dafang county (105°55′ E, 27°13′ N, 1500 m a.s.l.), Bijie, Guizhou province, one of the major cultivation areas of *G. elata* in China. The climate is subtropical humid monsoon with an average temperature of 12.5℃ and mean precipitation of 1, 100 mm. The trial began with *G. elata* cultivation in October 2016 and harvested in November 2017. This was followed by immediate cultivation of *P. impudicus* which was harvested in November 2018. The experiment consisted of three treatments including CK (soil initially collected before cultivation), GE (soil collected from the *G. elata* at its harvest time), and PI (soil collected from the *P. impudicus* at its harvest time). Three replicate samples from each treatment were collected at the harvest time. Detailed cultivating and sampling information as described below.

### Cultivation of *G. elata* and soil sampling

The trial was performed in a single field site that was newly explored to ensure uniformity. Three plots adjacent to each other, which were regarded as three biological replications, were chosen to dig holes for cultivation of *G. elata*. The holes were dug with sizes of 80 cm × 30 cm × 30 cm (length × width × depth) for each. After that, soil samples on the bottom of the hole (referred to as CK treatment) were initially collected by using five points sampling method. To cultivate *G. elata*, the bottom of the hole was carpeted with pieces of chestnut wood (size: 15 cm × 8 cm) that were fully soaked with water and infected with *A. gallica*. Subsequently, both sides of each chestnut wood were placed with two pieces of juvenile tubers (*G. elata* seeds) of *G. elata* in uniform sizes. The juvenile tubers of *G. elata* were then covered with twigs and leaves infected with *A. gallica* to maintain humidity at the growing season. Finally, soil was filled back into the holes and covered with twigs or leaves [12]. Considering that the growth of *G. elata* depended on the symbiotic association with *Armillaria*, and *Armillaria* species obtain nutrients from wood and require no fertilizers from the environment, no fertilizers were used in this field trial. The trial site is located under forest, and the twigs or leaves were used to keep humidity for the growth of *G. elata*. In this way, artificial watering was only conducted when the temperature was over 30℃ for more than 1 week.

During harvesting, topsoil, leaves, and bulk soil were carefully removed, then soil adhering to the *G. elata* (referred to as GE treatment) was collected, sieved (2 mm) and thoroughly homogenized within one hole. There were three biological replications for GE treatments. Each soil sample was divided into three portions for measurements: one portion with approximately 1 g was immediately frozen in liquid nitrogen and kept at − 80℃ until DNA extraction; another portion was stored at 4℃ for the measurement of soil ammonium nitrogen (NH_4_^+^-N) and nitrate nitrogen (NO_3_-N), and the remaining soil was air-dried prior to determination of soil properties.

### Cultivation of *P. impudicus* and soil sampling

The commercial strains used for *P. impudicus* cultivation were provided by Guizhou Wumeng Fungi Industry Co., Ltd. The strains originated from spores collected from the wild. After the purification of the cultures, the hyphae were maintained and subcultured on 2% (w/v) malt extract agar and kept at 23–25℃. The numbers of subculture were less than 5 generations, and the high-quality hyphae with strong and fast growth were selected as cultivation strains. Sixty to ninety days before field cultivation, the hyphae were inoculated to inoculum bags which contained sawdust (80%), wheat bran (7%), maizena (6%), soybean meal (4%), lime and gypsum (2%), sugar, magnesium sulfate, and potassium dihydrogen phosphate (1%) as the main nutrient materials. All these materials were mixed evenly, soaked with water to a final moisture of 60–70%, and subjected to autoclaving before inoculation. After inoculation, the inoculum bags were kept at 23℃ in a dark culturing chamber used for the cultivation of *P. impudicus*.

The *P. impudicus* was cultivated in the holes where the *G. elata* was harvested. To form a cultivation “bed” for *P. impudicus*, chestnut wood with small sizes (3 cm × 5 cm) were put in the middle of the gaps between the big chestnut wood cultivated for *G. elata*. Then, the inoculum bags filled with *P. impudicus* hypha were chopped into chunks with a diameter of 3∼5 cm. The placement distances of strain chunks were 5 cm, and the distance between rows was 10 cm. The following procedures were in accordance with cultivation of *G. elata* by filling the holes with soil, twigs or leaves. Watering only conducted when the temperature exceeded 30℃ for more than 1 weeks.

The harvest time was during October to November in the following year, when the “eggs” or fruit body of *P. impudicus* were come up out of the soil. To sample, the “eggs” or fruit body of *P. impudicus* were carefully removed from soil. Then, the soil surrounding the *P. impudicus* (referred to as PI treatment) was sampled and divided into 3 portions for measurements as described above.

### Measurements of soil biochemical properties

Carbon dioxide (CO_2_)-free water was used to for pH measurement. After mixing 2 g air-dried soil in 10 mL deionized water and letting this mixture to settle for 30 min, we measured the pH of each sample using a PHS-25 pH meter (Mettler Toledo, Giessen, Germany). Soil total nitrogen (TN) content was measured using the Kjeldahl method. Ammonium-nitrogen (NH_4_^+^-N) and nitrate-nitrogen (NO_3_-N) were extracted with fresh soils using 2 M potassium chloride (1:10), and their concentrations were measured using a continuous-flow analyzer (San++, Skalar, Netherlands).

Available manganese, zinc, and copper were extracted using diethylene triamine pentaacetic acid-triethanolamine-calcium chloride (DTPA-TEA-CaCl_2_, pH 7.3), while exchangeable potassium, calcium, and magnesium were extracted using ammonium acetate (pH 7.0). Contents of available elements and exchangeable cations were measured using atomic absorption spectrophotometry (AA-6880, Shimadzu, Japan). Humus was extracted using a mixture of 0.1 M sodium pyrophosphate and 0.1 M sodium hydroxide, and humus content was determined using the potassium dichromate heating oxidation-volumetric method.

### Soil DNA purification and Illumina MiSeq sequencing

Microbial DNA was extracted using the E.Z.N.A.® soil DNA Kit (Omega Bio-tech, Norcross, GA, U.S.). For the Illumina Miseq sequencing, the 16 S rRNA genes were amplified with primers 338 F (5′-ACTCCTACGGGAGGCAGCAG-3′) and 806R (5′-GGACTACHVGGGTWTCTAAT-3′), and the ITS rRNA genes were amplified with primers ITS1F (5′-CTTGGTCATTTAGAGGAAGTAA-3′) and ITS2R (5′-GCTGCGTTCTTCATCGATGC-3′) with a 12 nt unique barcode at the 5′ end. The assays were performed in a 20 µL mixture containing 10 µL of SYBR® Premix Ex Taq (Tli RNaseH Plus, 2×, Takara Bio, Japan), 1 µL of forward primer and 1 µL of reverse primer at 10 mM, 7 µL Milli-Q water, and 1 µL of 10-fold diluted DNA. The initial denaturation was at 95 °C for 2 min, followed by 25 cycles of denaturation at 95 °C for 30 s, annealing at 55 °C for 30 s, and extension at 72 °C for 45 s, with a final extension at 72 °C for 10 min [21]. The PCR products were isolated and extracted from a 2% agarose gel, then purified (TruSeqTM DNA Sample Prep Kit). Further, the purified amplicons were pooled in equimolar and paired-end sequenced (2 × 300) on an Illumina MiSeq platform (Illumina, San Diego, USA) according to the standard protocols by Majorbio Bio-Pharm Technology Co. Ltd. (Shanghai, China).

### Processing of illumina MiSeq sequencing raw data

The QIIME software package (version 1.9.1) was used to analyze the Illumina MiSeq sequencing raw data [22]. The reads were quality trimmed by discarding quality scores less than 20 and sequence lengths less than 400 bp. In total, 193, 498, 292 high quality reads for 16 S rRNA genes and 183, 686, 532 high quality reads for ITS rRNA genes were obtained. After preprocessing all the reads, the numbers of sequences among different samples ranged from 30, 414 to 42, 466 in 16 S rRNA genes and 49, 543 to 62, 089 in ITS rRNA genes. We randomly selected sequences based on the minimum reads for all the samples before further analysis. The operational taxonomic units (OTUs) were defined at 97% similarity level using Usearch (version 11 http://drive5.com/uparse/) with the remaining and unique sequences. The taxonomic identity of the phylotypes was determined by the Ribosomal Database Project (RDP) Classifier (version 2.13 http://sourceforge.net/projects/rdpclassifier/) at a confidence threshold of 70%.

### Analysis of bioinformatics data

#### β-diversity and Venn analysis

Based on the calculated Bray-Curtis distance, principal coordinate analysis (PCoA) and hierarchical clustering analysis (HCA) were performed to investigate the variation of microbial community structure among treatments. To assess the difference in the microbial community composition variations, permutational multivariate ANOVA (PERMANOVA) was conducted using the anosim function in the R software package vegan (version 2.6-4).

The Venn diagrams were constructed to visualize shared and unique OTUs among samples. The core OTUs existing in all soil samples were selected and visualized as microbial community pie charts on the platform Majorbio (www.majorbio.com).

### Bipartite network analysis

Based on the pairwise spearman method and correlation coefficient with threshold value *r* ≥ 0.5, the bipartite network analysis on genus level of bacterial and fungal were calculated respectively at *p* < 0.05 significant level. Then the network was visualized by Cytoscape software (version 3.7.1) by using the treatments as source nodes and the genera as target nodes [23], with edges corresponding to positive associations of genera under different cultivation management.

### Canonical correspondence analysis

A mantel test was conducted to test the significance of the effect of the soil physicochemical properties [pH, SOC (soil organic carbon), TN, NH_4_^+^-N, NO_3_-N, AP (available phosphorus), AK (available potassium), and C/N (ratio of soil carbon to nitrogen)] on the bacterial and fungal community composition with a Euclidean distance dissimilarity matrix. The parameters identified as significant (*p* ≤ 0.05) in the mantel test were used to perform a canonical correspondence analysis (CCA) to assess the major environmental factors that determined bacterial and fungal community composition at the OTU level. Both mantel test and CCA analysis were performed using the vegan package (version 2.6-4) in R software (version 4.1.2) for Windows [24].

### Soil bacterial and fungal composition and heatmap

The significance in relative abundance at the genus level among different soil samples was tested using the one-way analysis of variance (one-way ANOVA) and Tukey-Kramer test at a 0.05 significant level. Dominant genera with significant changes were presented in figures. Subsequently, the association of dominant genera with significant changes integrated with soil chemical properties were analyzed by performing a heatmap. The heatmap was constructed with the Psych, Pheatmap, and Reshape2 packages in R software (version 4.1.2) for Windows [24].

### Statistical analysis

We performed one-way ANOVA to assess the significance of differences in α diversity of soil bacterial and fungal communities, soil biochemistry, available elements, exchangeable cations, and humus variations among different soil samples. Differences associated with *p* < 0.05 based on Duncan’s multiple range test were defined as significant. All analyses were performed using IBM SPSS Statistics 26.0 (IBM, Chicago, IL, USA).

## Results

### Cultivations of *G. elata* and *P. impudicus* affected soil biochemical properties

All soil samples, including the control, were acidic (Table 1), the CK (soil collected from fallow field) demonstrating the lowest pH value. The cultivation resulted in the increase in soil pH value in GE (soil adhering to the *G. elata*) and PI (soil surrounding the *P. impudicus*) treatments. A similar trend was also found in soil available elements and exchangeable cations, such as available manganese and copper, soil exchangeable potassium and magnesium (Fig. S2). There was no significant (*p* = 0.209) difference in NH_4_^+^-N concentrations between CK and GE. The highest NH_4_^+^-N concentrations was observed in PI sample. However, GE had significantly high NO_3_-N concentration than CK and PI samples did (*p* < 0.05). Total N content did not differ significantly among CK, GE and PI (*p* = 0.171, Table 1). The PI treatment resulted in the highest available phosphorus (AP), and significantly higher than that in CK treatment (*p* < 0.05), while there was no significant difference between CK and GE in AP content (*p* = 0.084).

**Table 1:** Changes of soil biochemical properties in response to different cultivation management systems

Treatments	pH	TN (g·kg^− 1^)	NH_4_^+^-*N* (mg·kg^− 1^)	NO_3_-*N* (mg·kg^− 1^)	AP (mg·kg^− 1^)
CK	4.44 ± 0.10 c	1.63 ± 0.73 a	19 ± 7.79 b	8.48 ± 2.12 b	3.73 ± 0.31 b
GE	5.76 ± 0.03 a	2.50 ± 0.44 a	7.65 ± 0.35 b	22 ± 2.90 a	6.38 ± 0.16 ab
PI	4.88 ± 0.02 b	0.86 ± 0.34 a	71 ± 6.36 a	13.4 ± 2.27 b	9.00 ± 1.53 a

The different letters within a column represent significant differences among the treatments (*p* ≤ 0.05), determined by one-way ANOVA and Duncan’s multiple range test. The data are the means of 3 replicates ± standard error. Abbreviation: CK, GE, and PI represent soil samples collected from fallow field (control), soil adhering to the *G. elata*, and soil surrounding the *P. impudicus*, respectively.

### Soil bacterial and fungal α- and β-diversities responding to cultivation of *G. elata* and *P. impudicus*

The Chao 1 and Shannon indexes of bacterial and fungal communities were higher in GE and PI, compared to that in CK (*p* < 0.01, Table 2). There was no significant difference in the richness of microbial community between GE and PI as indicated by Chao 1 index (*p* = 0.707 for bacteria, *p* = 0.056 for fungi). In contrast, the Shannon index of bacterial and fungal communities in GE was significantly higher than that in CK and PI (*p* < 0.05). These results implied that either cultivation of *G. elata* or *P. impudicus* would greatly influence the diversity of soil bacterial and fungal communities.

**Table 2:** Soil microbial community diversity indices in different soil samples

Treatments	Chao 1	Shannon index		Chao 1	Shannon index
	bacterial community			fungal community	
CK	1133 ± 13.7 b	5.44 ± 0.02 c		743 ± 40.7 b	3.91 ± 0.02 c
GE	1502 ± 32.1 a	6.00 ± 0.05 a		1412 ± 59.0 a	4.86 ± 0.02 a
PI	1516 ± 24.6 a	5.89 ± 0.01 b		1249 ± 44.4 a	4.69 ± 0.03 b

The different letters within a column represent significant differences among the treatments (*p* ≤ 0.05), determined by one-way ANOVA and Duncan’s multiple range test. The data are the means of 3 replicates ± standard error. Abbreviation: CK, GE, and PI represent soil samples collected from fallow field (control), soil adhering to the *G. elata*, and soil surrounding the *P. impudicus*, respectively.

**Fig. 1:**
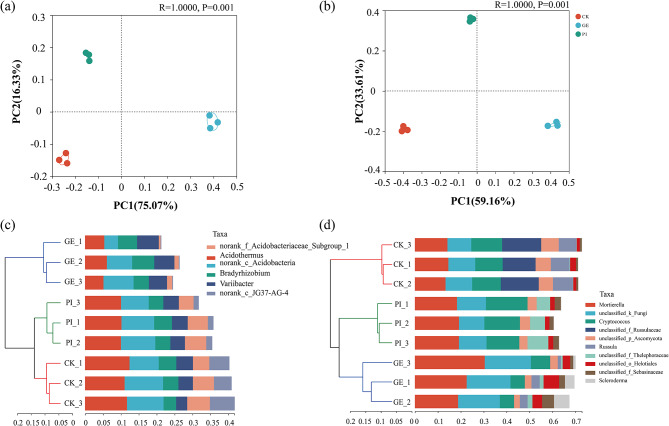
Principal coordinate analysis (PCoA) on OTU level and hierarchical clustering analysis (HCA) on genus level, both were calculated based on the Bray-Curtis distance. Panels **a**, and **b**, the PCoA analysis of bacterial and fungal communities; panels **c**, and **d**, the HCA analysis of bacterial and fungal communities. Abbreviation: CK, GE, and PI represent soil samples collected from fallow field (control), soil adhering to the *G. elata*, and soil surrounding the *P. impudicus*, respectively

Overall, the Bray-Curtis PCoA analysis showed that the bacterial and fungal community structure in GE and PI were greatly separated from CK (Fig. 1a, b), which indicated that cultivation of *G. elata* and *P. impudicus* had significant impacts on soil microbial community structure (*p* < 0.05). The tree diagram showed that group of CK and PI joined together and shared long distance with GE, and HCA result indicated that the taxa of CK and PI were more similar with each other in bacterial community (the HCA distance for CK and PI was 0.13, while that value for CK, PI and GE was 0.17, Fig. 1c) and more abundant than that in GE. Whereas, the tree diagram and HCA results of fungal community indicated that group or taxa in PI and GE were more similar than those in CK (the HCA was 0.12, Fig. 1d). Specifically, some taxa like *unclassified_f_Russulaccac* were abundantly present while others like *Sclerodema*, *unclassified_f_Thelephoraceae* were lost or less abundant in CK when compared with GE and PI.

**Fig. 2:**
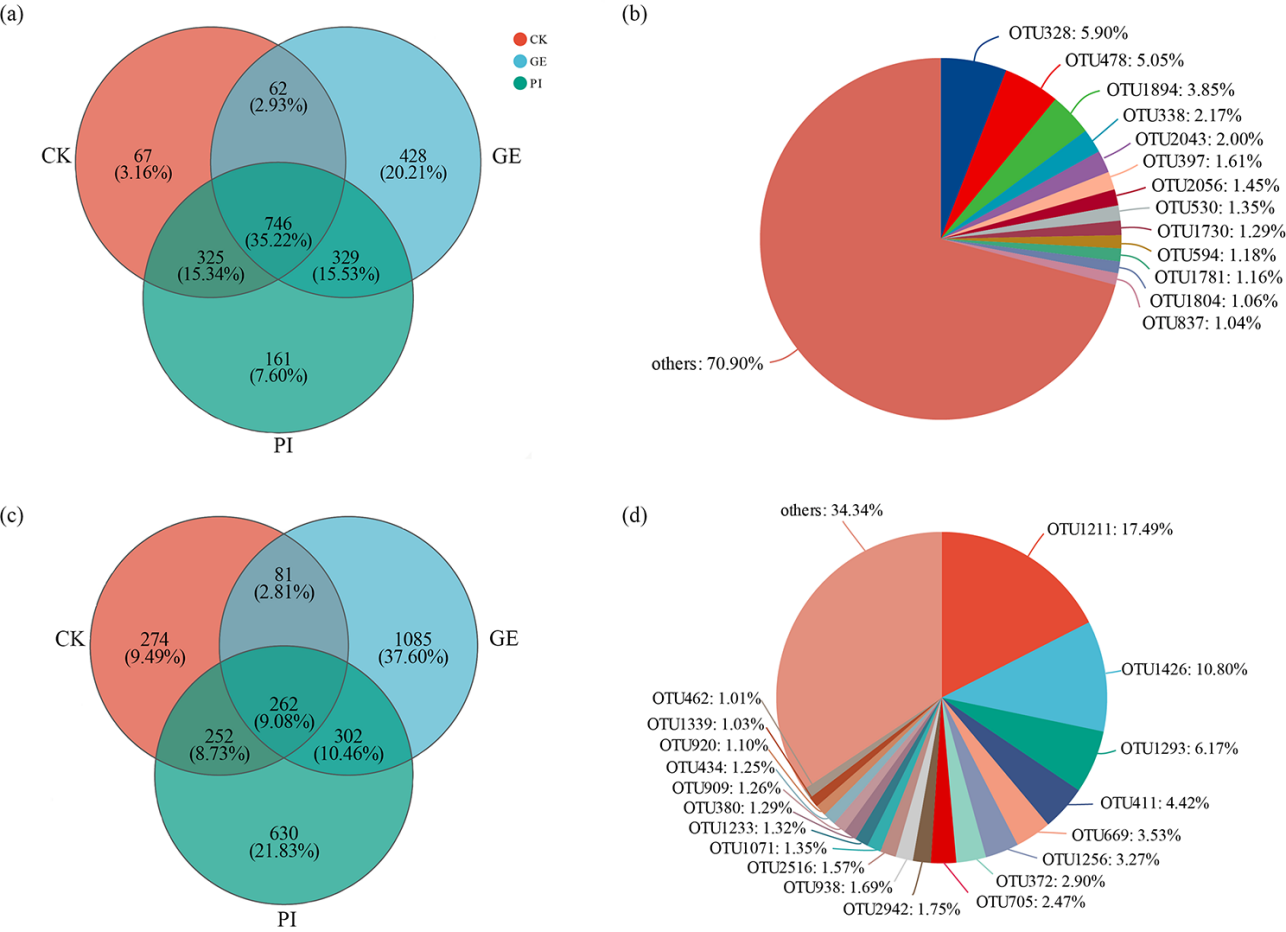
Venn diagram and pie charts of different soil samples. Panels **a**, and **b**, the Venn diagrams and pie charts of bacterial community on OTU level; panels **c**, and **d**, the Venn diagrams and pie charts of fungal community on OTU level. Abbreviation: CK, GE, and PI represent soil samples collected from fallow field (control), soil adhering to the *G. elata*, and soil surrounding the *P. impudicus*, respectively

The shared bacterial OTU numbers within CK and PI were 325 (15.34%), within GE and PI were 329 (15.53%), while within CK and GE were 62 (2.93%) (Fig. 2a). The shared OTU numbers within CK, GE and PI were 746 (35.22%) in which the others comprised 70.90% followed by OTU 328 (5.9%), OTU 478 (5.05%), and OTU 1894 (3.85%) (Fig. 2b). Moreover, the shared fungal OTU numbers within CK and PI, GE and PI, CK and GE were 252 (8.73%), 302 (10.46%), and 81 (2.81%) respectively (Fig. 2c). The shared OTU numbers within CK, GE and PI were 262 (9.08%) in which OTU 1211, OTU 1426, and OTU 1293 took up 17.49%, 10.8%, and 6.17% which occupied the first three compositions besides the others (34.34%) (Fig. 2d). Moreover, there are more peculiar OTU numbers in GE and PI compared to that in CK (Fig. 2a, c), especially in the soil fungal community.

### The networks of soil bacterial and fungal communities became more complex in response to cultivation of*G. elata* and *P. impudicus*

**Fig. 3:**
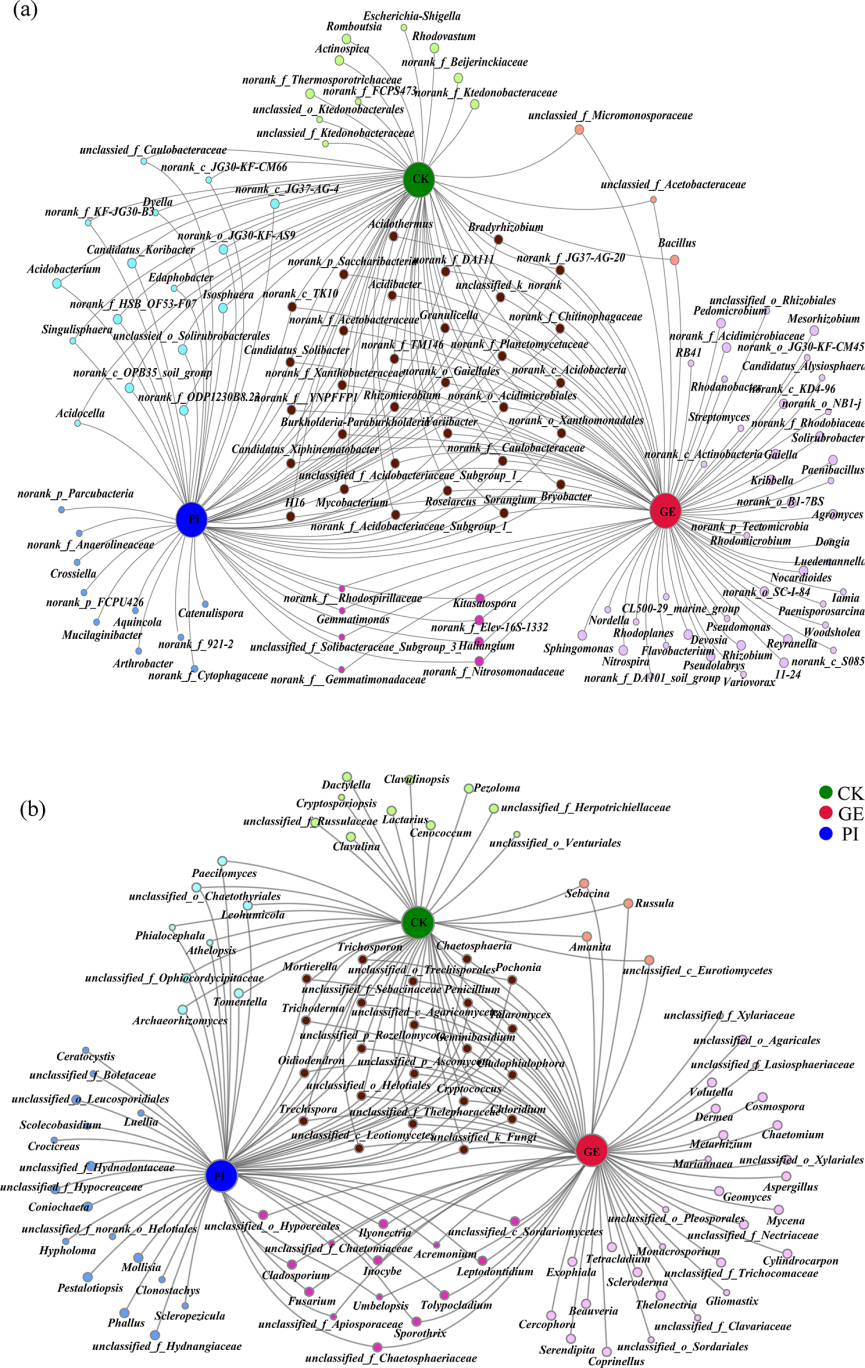
Co-occurrence analysis of bacterial (**a**) and fungal (**b**) communities under different cultivation management systems

The bipartite networks of bacterial and fungal communities, respectively, were constructed to visualize the associations between genera and the different planting strategies or cultivation management (Fig. 3). The closeness centralities of CK, GE, and PI in bacteria were 0.57, 0.83, and 0.71 (Fig. 3a), and those in fungi were 0.49, 0.71, and 0.61, respectively (Fig. 3b).

Of the presented genera, 44.4% were most strongly associated with only one system in bacteria (Fig. 3a), and 53.8% were most strongly associated with only one system in fungi (Fig. 3b), confirming the basic distinctness of these genera in all managements. Approximately 32.1% of the significant genera were most strongly driven by GE, whereas another 7.0% were associated with PI in bacterial network. In fungal network, 27.9% of the significant genera were most strongly driven by GE, while 16.3% were associated with PI. There were 19.0% and 25% of the significant genera that were associated with two cross-combinations (genera nodes associated with two treatments) in bacterial and fungal networks, while the three cross-combinations (genera nodes associated with three treatments) accounted for 22.5% and 21.2% in bacterial and fungal networks, respectively.

### Effect of cultivation of *G. elata* and *P. impudicus* on soil bacterial and fungal compositions

**Fig. 4:**
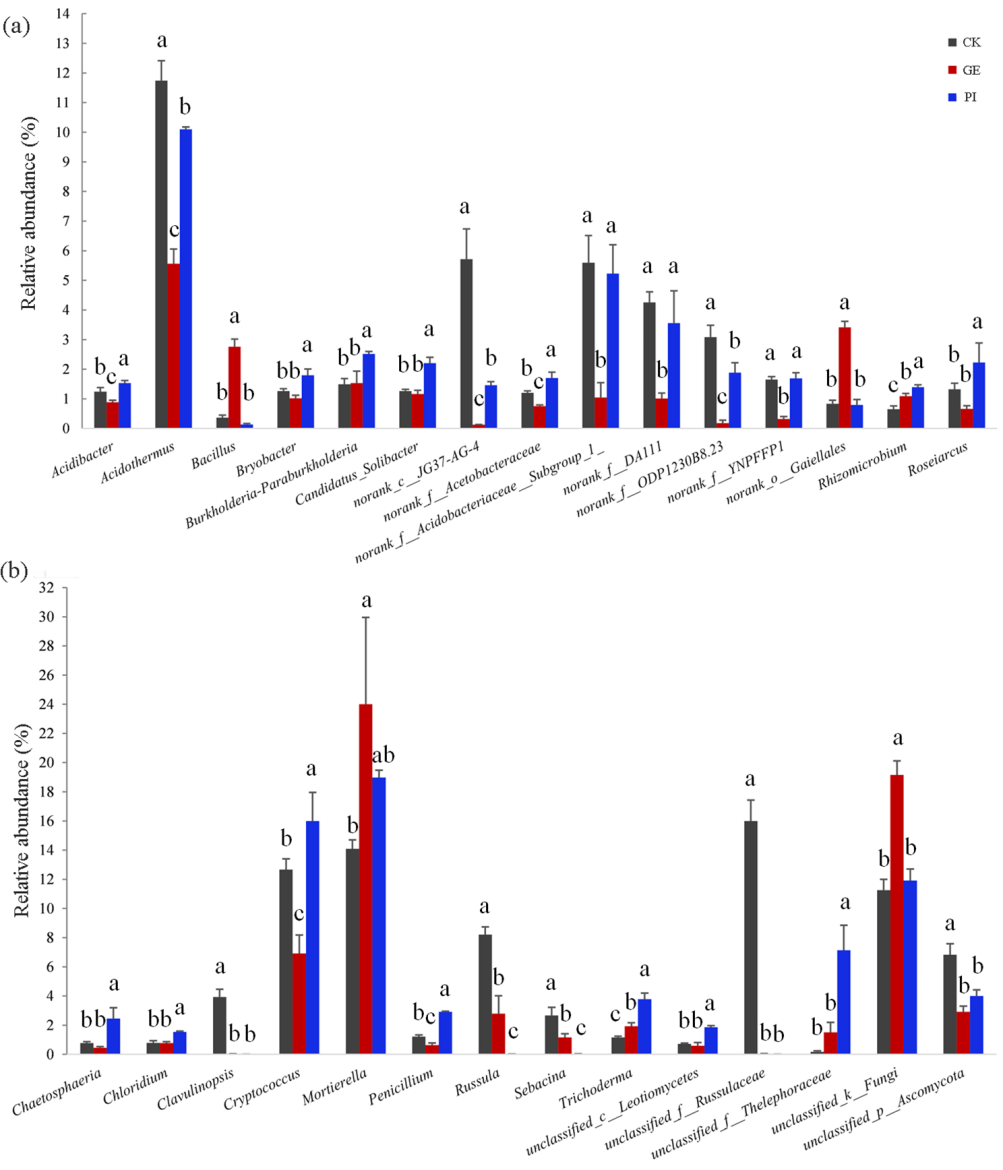
Relative abundances of bacterial (**a**) and fungal (**b**) genera under different cultivation management systems. Abbreviation: CK, GE, and PI represent soil samples collected from fallow field (control), soil adhering to the *G. elata*, and soil surrounding the *P. impudicus*, respectively. Lowercases a, b, and c in figures indicate significant differences among different soils (CK, GE and PI), determined by one-way ANOVA and Duncan’s multiple range test (*p* < 0.05)

For bacterial genera (Fig. 4a), the relative abundances of *Bacillus* and *norank_o_Gaiellales* were significantly increased (*p* < 0.05), while the relative abundances of *Acidibacter*, *Acidothermus*, *norank_c__JG37-AG-4*, *norank_f__Acidobacteriaceae__Subgroup_1_*, *norank_f__DA111*, *norank_f__ODP1230B8.23*, and *norank_f__YNPFFP1* were significantly decreased in GE compared to CK (*p* < 0.05). Interestingly, PI helped to increase the relative abundances of genera which GE decreased before. Alternatively, the relative abundances of some genera, like *Bryobacter*, *Burkholderia-Paraburkholderia*, *Candidatus_Solicaber*, and *Roseiarcus* showed no significant difference between GE and CK. However, compared with GE and CK, the relative abundances of these genera in PI were significantly increased (*p* < 0.05).

As for fungal genera (Fig. 4b), the relative abundances of *Clavulinopsis*, *Russula*, *Sebacina*, *unclassified_f_Russulaceae*, and *unclassified_p_Ascomycota* were significantly decreased in GE and PI compared to CK (*p* < 0.05). However, the relative abundance of *Mortierella* and *unclassified_k_Fungi* were greatly increased in GE compared with CK. No significant differences were found in relative abundances of *Chaetosphaeria*, *Chloridium*, *unclassified_c_Leotiomycetes*, and *unclassified_f_Russulaceae* between GE and CK, however, their abundances were greatly increased in PI samples (*p* < 0.05).

### Association of bacterial and fungal communities with soil biochemical factors

**Fig. 5:**
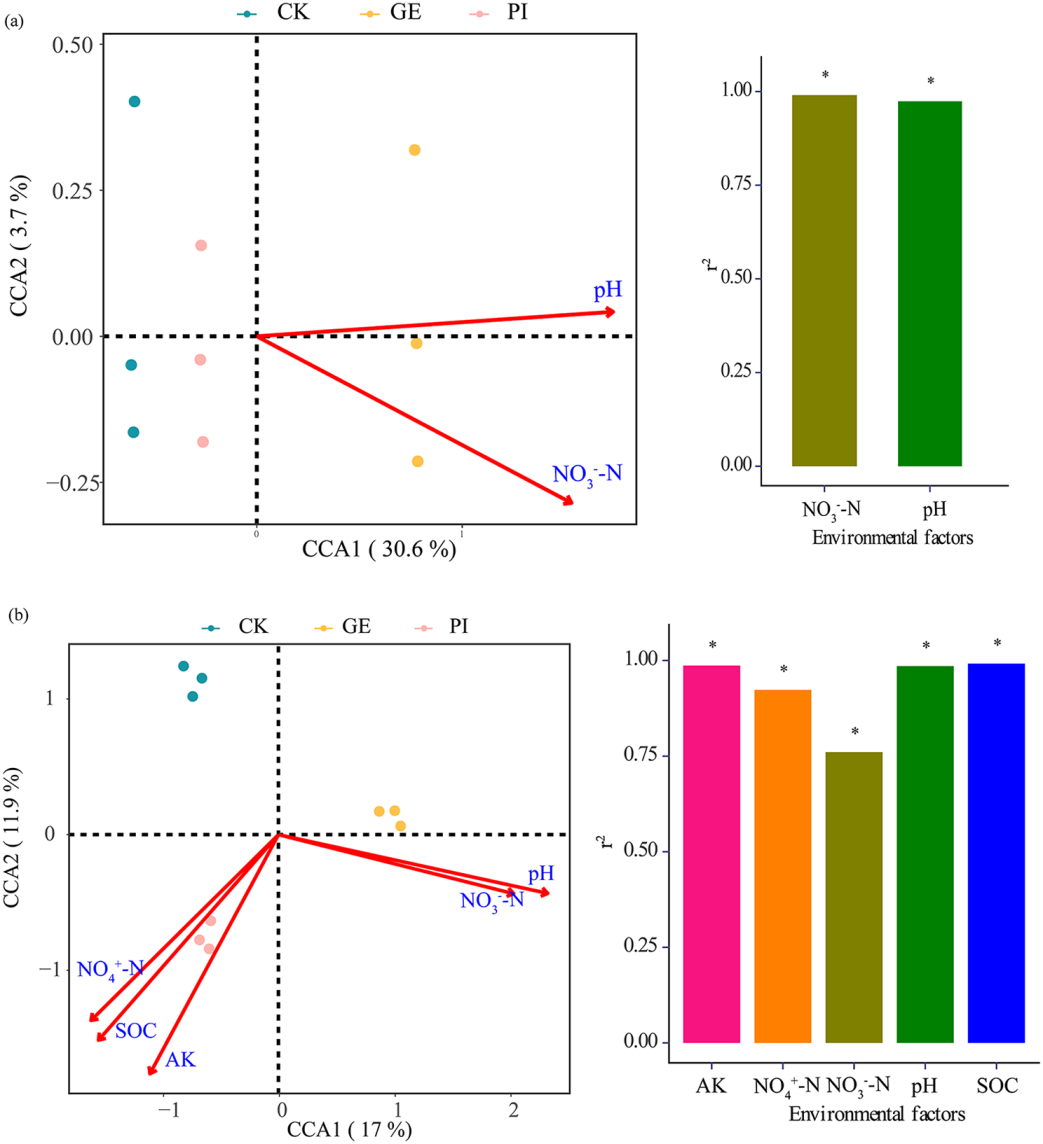
Canonical correlation analysis (CCA) of environmental characteristics (arrows) and MiSeq data (symbols) for (**a**) soil bacterial communities and (**b**) soil fungal communities under different cultivation management systems. Abbreviation: CK, GE, and PI represent soil samples collected from fallow field (control), soil adhering to the *G. elata*, and soil surrounding the *P. impudicus*, respectively

The canonical correlation analysis (CCA) was performed to reveal the driving factors for developing soil bacterial and fungal communities on the genus level (Fig. 5). The result showed that the changes in bacterial communities were significantly correlated with soil pH and NO_3_-N (*p* < 0.05, Fig. 5a), while the fungal communities were significantly affected by soil factors such as pH, NO_3_-N, NH_4_^+^-N, SOC, and AK (*p* < 0.05, Fig. 5b). The first axis in the CCA results explained 30.6% and 17.0% of the bacterial and fungal community variations, respectively, which suggested a remarkable restrictive rate of CCA1 to microbial community and soil properties. The results of CCA indicated that soil properties, especially soil pH value might impose significant impacts on soil bacterial and fungal communities in *G. elata* or *P. impudicus* cultivation.

**Fig. 6:**
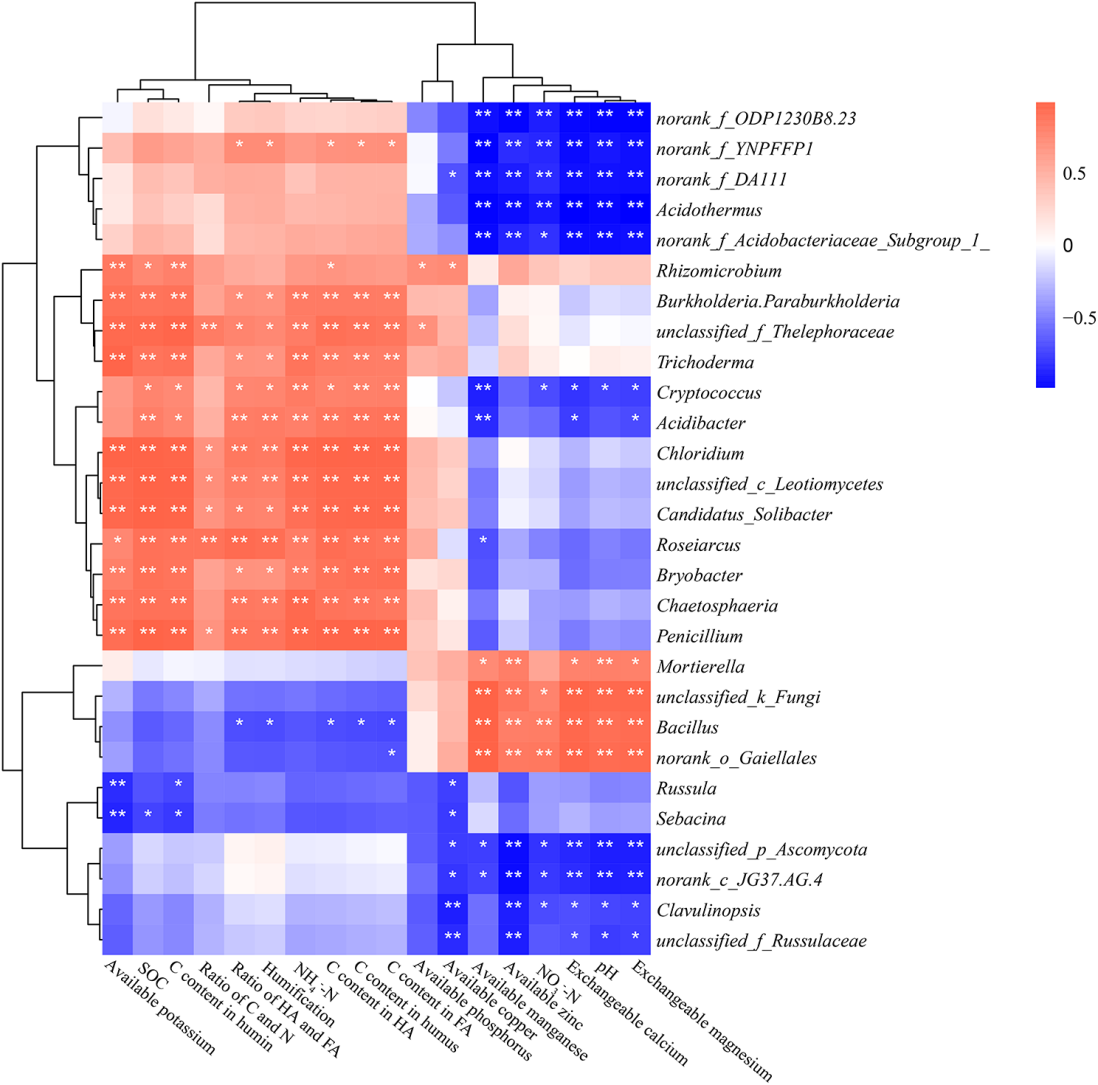
Heatmap based on Pearson correlation analysis between the physicochemical properties and the genera (including both bacteria and fungi) under different cultivation management systems. Significant differences (p-FDR < 0.05) to planting strategies indicated by “*”

To further analyze the correlation between the soil physicochemical properties and the relative abundance of soil microorganisms, Pearson analysis correlation was conducted based on a heatmap (Fig. 6). In general, the C/N ratio is an important index for indicating the nutritional balance, but only 6 taxa had significant correlations with it. Overall, the pH and NO_3_-N had strong correlations with the bacteria and fungi detected. Among all these 28 genera, 50% and 42.9% demonstrated significant correlations with pH and NO_3_-N, respectively. Microelements such as exchangeable magnesium and calcium, available zinc, and manganese also played an important role in controlling the relative abundances of microorganisms. They generally showed strong correlations with 13–15 genera, which accounted for 46.4–53.6% of all the genera provided. In addition, the carbon related soil factors such as SOC, C content in humin and humus, showed a strong positive correlation with the genera like *Rhizomicrobium*, *Burkholderia-Paraburkholderia*, *unclassified_f_Thelephoraceae*, *Trichoderma*, *Cryptococcus*, *Acidibacter*, *Chloridium*, *unclassified_c_Leotiomycetes*, *Candidatus_Solibacter*, *Roseiarcus*, *Bryobacter*, *Chaetosphaeria*, and *Penicillium*. These findings suggest that chemical properties such as soil pH, NO_3_-N, or soil C storage which would be regulated by *G. elata* or *P. impudicus* cultivations, may play a crucial role in regulating the relative abundance of soil microorganisms.

## Discussion

Monoculture management has substantially reduced soil biodiversity as well as agroecosystem functions not only in agricultural crops but also medicinal herbs. Proper agricultural management practices, such as crop rotation, may promote soil biodiversity [25]. Unlike plants that can influence soil microbial activity and diversity through root exudations, it is more likely that the saprotrophic fungi like *Armillaria* species and *P. impudicus* influence their microenvironment through secretions and suppress the growth of some fungi by competing with niche status in the soil.

The present study found that cultivation of either *G. elata* or *P. impudicus* would result in the absence of fungi *unclassified_f_Russulaccac* (Fig. 1d), which is different from previous studies reporting that farming induced higher relative abundances of saprotrophic fungi [26]. Being as saprotrophic fungi, it is possible that the nutrients required by *A. gallica* (the symbiont of *G. elata*) and *P. impudicus* were partially in common with *unclassified_f_Russulaccac*, and that the strong inhibiting tendency of *Armillaria* and *Phallus* members restrained the growth of saprotrophic fungi like *unclassified_f_Russulaccac* and others [27]. Additionally, *P. impudicus* impelled the soil bacterial taxa classification to be more similar with that in fallow land, while closely associating with fungal taxa in *G. elata* cultivation (Fig. 1b, d). These findings suggested that *P. impudicus* contributed and shaped the taxonomic distribution patterns of different types of microorganisms in this trial.

In addition, the relative abundance of some potentially beneficial organisms, such as *Candidatus_Solibacter* and *Penicillium*, significantly increased in soil surrounding the *P. impudicus* compared with that in fallow land and soil adhering to the *G. elata* (Fig. 4). The members of *Candidatus_Solibacter* were reported to be related to the degradation of potential soil allelochemicals [28]. Although some species of *Penicillium* can cause crop diseases, most of them have been discovered as beneficial fungi which can control plant diseases by inhibiting the growth of pathogenic organisms [29]. The relative abundance of other microbes such as *Trichoderma*, showed the same trend with *Candidatus_Solibacter* and *Penicillium*, implying the sensitivity of these potentially beneficial fungi to tillage practices, especially the cultivation of *P. impudicus*. This indicated that the *P. impudicus* may recruit potentially beneficial genera to adapt to the environment. Results of these findings could have important implications for our understanding of the role of appropriate targets in manipulating beneficial organisms to improve the environment in artificial cultivation of *G. elata*.

There is a consensus that cultivation of *G. elata* does not need fertilizers and that its nutrient requirements are fulfilled by its symbiont fungi, *Armillaria*, which are well-known as wood decomposers [30]. If this was the case, how could it happen that some soil biochemical properties changed with cultivation of *G. elata* and *P. impudicus*? The possible explanations for these observations in this study may be in partial attributed to some soil functional genera that were activated by simple compounds degraded by enzymes that were secreted by *Armillaria* members [31, 32] or the direct secretions of *A. gallica*.

For instance, the relative abundance of *Bacillus* significantly increased in the cultivation of *G. elata*, and sequentially decreased in the cultivation of *P. impudicus* (Fig. 4), which was in accordance with the changes of NO_3_-N and microelements (Table 1 & Fig. S2). The response of *Bacillus* in our study coincided with a previous study which indicated that the rhizomes of *G. elata* may specifically attract high nitrogen-dependant bacteria such as *Bacillus* and others [12]. It has been reported that elements such as potassium, zinc, magnesium, and calcium are easily accessible to soil microorganisms and are prone to change the activity and composition of the microbial community [33]. The genus *Bacillus* is capable of producing antibiotic substances and certain species have the capacity to fix nitrogen [34]. It is likely that the microelements released by *A. gallica* through secretions presumably induced the enrichment of *Bacillus* in cultivation of *G. elata* [35], then the soil nitrogen was solubilized into available form by the enriched *Bacillus* members. Evidence is also provided by the heatmap which revealed that elements such as NH_3_-N, available zinc, exchangeable magnesium, and calcium were in strong positive correlation with *Bacillus* (Fig. 6). If so, these results seem to suggest that the distribution of this genus and others like *norank_o_Gaiellales*, *unclassified_k_Fungi*, and *Mortierella* are in a particular trophic niche, and will rather be influenced and characterized by cultivation systems [36].

Additionally, numerous studies have demonstrated the ability of *Armillaria* species, as well as *P. impudicus* in accumulating available nutrients such as phosphorus from soil [37, 38], even though they are all saprotrophic wood-decaying Basidiomycotas. Therefore, the redistribution of available nutrients to soil by *Armillaria* and *P. impudicus* species may also possibly contribute to changes in the soil’s biochemical properties.

According to previous studies, soil biochemical properties, notably pH, are considered as major drivers of determining the soil bacterial diversity and composition [39]. Examining the effects of specific soil attributes, for instance the soil pH, on microbial taxa should help to reveal what drives their population changes [40]. The cultivation of *P. impudicus* decreased soil pH, which cultivation of *G. elata* increased before (Table 1). The observed increase of pH in soil associated with *G. elata* might be caused by the abundance of alkaline rhizomorph exudates released by *A. gallica* [35]. The CCA analysis suggested that soil pH integrated with soil nutrition had a strong correlation with microbial community changes in the cultivation of *G. elata* (Fig. 5). The truth is, the cultivation of *G. elata* and *P. impudicus* affected the abundances of several genera across several phyla, in which most of the genera were also associated with soil physicochemical properties, especially some soil taxa that were more sensitive to soil pH variations. For example, *Acidothermus*, an acidophilic genus, whose abundance initially decreased with the increase of pH in *G. elata* cultivation, then increased with the reduction of pH under cultivation of *P. impudicus* (Fig. 4a; Table 1), was also found to have strong correlation with soil pH (Fig. 6). This indicated that cultivation of *P. impudicus* helped to improve the environment for acidophilic genera in soil. In other words, the cultivation of *P. impudicus* can help maintain soil microbial composition by recovering soil nutrient equilibrium, which is also closely related to other soil microorganisms’ activities [41].

According to previous studies, *Armillaria* members normally assimilate soil carbon and significantly reduced carbon content in humus, especially levels of fulvic acid to obtain its growth materials [42]. However, *P. impudicus* was reported to have the ability to producing dense cross-linked networks of thick hyphae, and bonding soil particles into a coacervate [43]. Therefore, in our study, cultivation of *P. impudicus* was observed to increase the ratio of humic to fulvic acid, a measure of humification (Table S1). This implied that soils where *P. impudicus* was applied after *G. elata* had greater carbon storage capacity than soils where only *G. elata* was cultivated. It is commonly accepted that high C storage is vital for soil health and long-term ecosystem sustainability [44]. Considering that *G. elata* is normally cultivated in acidic soils [45, 46], and the *A. gallica* rhizomorphs typically grow well at a depth of 30 cm in soil with pH 4.3–4.6 [47], soil microenvironment re-optimized by cultivation of *P. impudicus* might become preferable for the growth of *G. elata* and its symbiont *A. gallica*.

In this study, several genera showed significantly higher relative abundance in soil associated with cultivation of *P. impudicus* (Fig. 4), which was consistent with the changes in soil humus composition and properties among different treatments (Fig. 6, Table S1). This indicated that, in addition to soil pH value, other soil parameters such as soil C sources, also contributed to the microbial community composition. The CCA result of fungal community strongly resembled and confirmed this observation (Fig. 5b). Similar observations were also made by previous authors who emphasized that SOC was essential in explaining the differences in the soil bacterial community structures in tropical agricultural soil [48]. Overall, the shift in patterns of soil microbial community composition and structure in soil associated with cultivation of *G*. *elata* and *P. impudicus* were related to soil physicochemical changes, which were mediated by the means of cultivation.

However, to conduct a comprehensive assessment on the effect of introducing *P. impudicus* into this *G. elata*-based soil system, it is necessary to conduct a more rational and long-term trial in future research. For example, addressing *G. elata* monoculture in parallel with *P. impudicus* cultivation, as well as cultivation systems sustained until the third year. Furthermore, the assessment needs to be extended to measure the secretions of *A. gallica* or *P. impudicus* at the harvesting period. This optimization and promotion of trial design will make it possible to assess the inferences of fungi secretions imposed on the biodiversity, which is closely related to the achievement of truly sustainable agriculture.

## Conclusion

Changes in the soil biochemical properties and soil microbial communities before and after cultivation of *G. elata* and *P. impudicus* were assessed and discussed in this study. The introduction of *P. impudicus* in the *G. elata* based soil, had an implication in improving soil quality by reversing *G. elata*-induced changes in soil pH, nutrient levels, and humus composition. The composition and diversity of soil microbial communities were greatly altered in response to the cultivation of *G. elata* and *P. impudicus*, especially some genera that have potentially beneficial functions in soil nutrient cycling, such as *Acidibacter*, *Acidothermus*, *Cryptococcus*, and *Penicillium*. By analyzing the correlation of soil microbial communities and chemical properties, a strong correlation of potentially beneficial genera with the soil pH and nutrients contents was observed. These results imply that cultivation of *P. impudicus*, compared to cultivation of *G. elata*, was likely to have a beneficial effect on plant health in the following growing season due to the rebalanced soil biochemical properties and the corresponding response in the soil microbial communities.

### Electronic supplementary material

Below is the link to the electronic supplementary material.

Supplementary Material 1

## Data Availability

Raw data of 16 S rRNA and ITS rRNA amplicon sequences supporting the findings of the present study are available in the Sequence Read Archive of NCBI under Bio Project accession PRJNA905858 and PRJNA905861, respectively.
