# Supplementary material for: Composition and diversity of soil microbial communities change by introducing Phallus impudicus into a Gastrodia elata Bl.-based soil
Source: BMC Microbiol. 2024 Jun 8;24:204. doi: 10.1186/s12866-024-03330-4 (PMC11161949; doi:10.1186/s12866-024-03330-4)
Supplement: Supplementary file 1 — Supplementary Material 1 [file 12866_2024_3330_MOESM1_ESM.docx]

Journal name: BMC Microbiology

**Changes in the composition and diversity of soil microbial communities by introducing *Phallus impudicus* into a *Gastrodia elata* Bl.-based soil**

Yanhong Wang^1,^ **^†^**, Jiao Xu^1,^ **^†^**, Qingsong Yuan^1^, Lanping Guo^2^, Gang Zheng^3^, Chenghong Xiao^1^, Changgui Yang^1^, Weike Jiang^1^, Tao Zhou^1, *^

^1^ Resource Institute for Chinese and Ethnic Materia Medica, Guizhou University of Traditional Chinese Medicine, Guiyang, China.

^2^ State Key Laboratory for Quality Ensurance and Sustainable Use of Dao-di Herbs, National Resource Center for Chinese Materia Medica, China Academy of Chinese Medical Sciences, Beijing, China.

^3^ Shanghai Tianyou Hospital Affiliated to Tongji University, Shanghai, China

**^†^**These authors have contributed equally to this work

*Correspondence: taozhou88@163.com

**
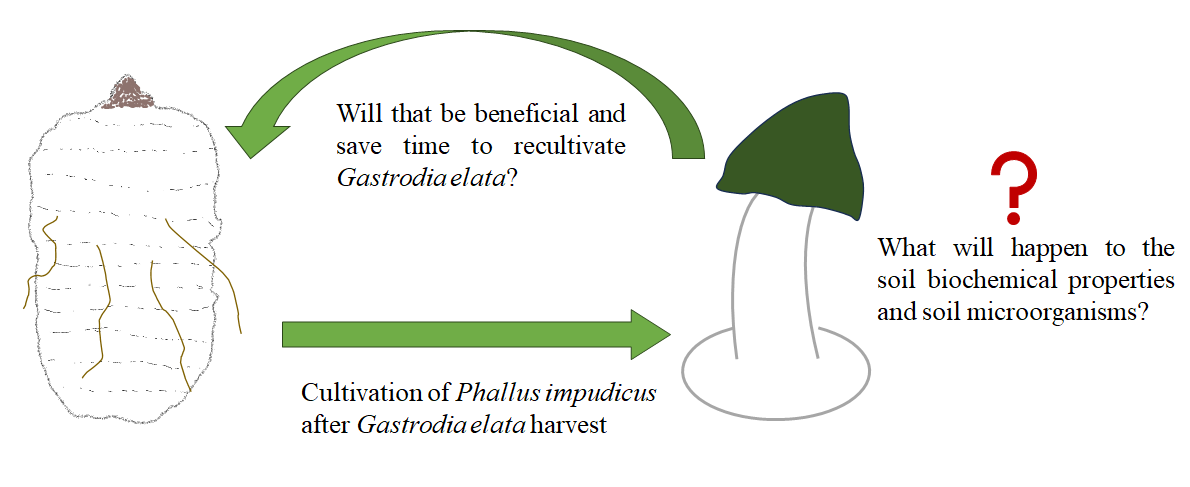
**

**Fig. S1.** The scheme of introducing *Phallus impudicus* to *Gastrodia elata* Bl.-based soil. Normally, successful replanting of *G. elata* requires leaving the land uncultivated for more than 3 years, investigation on soil biochemical and organisms were conducted to elucidate the effectively improving strategy.

**
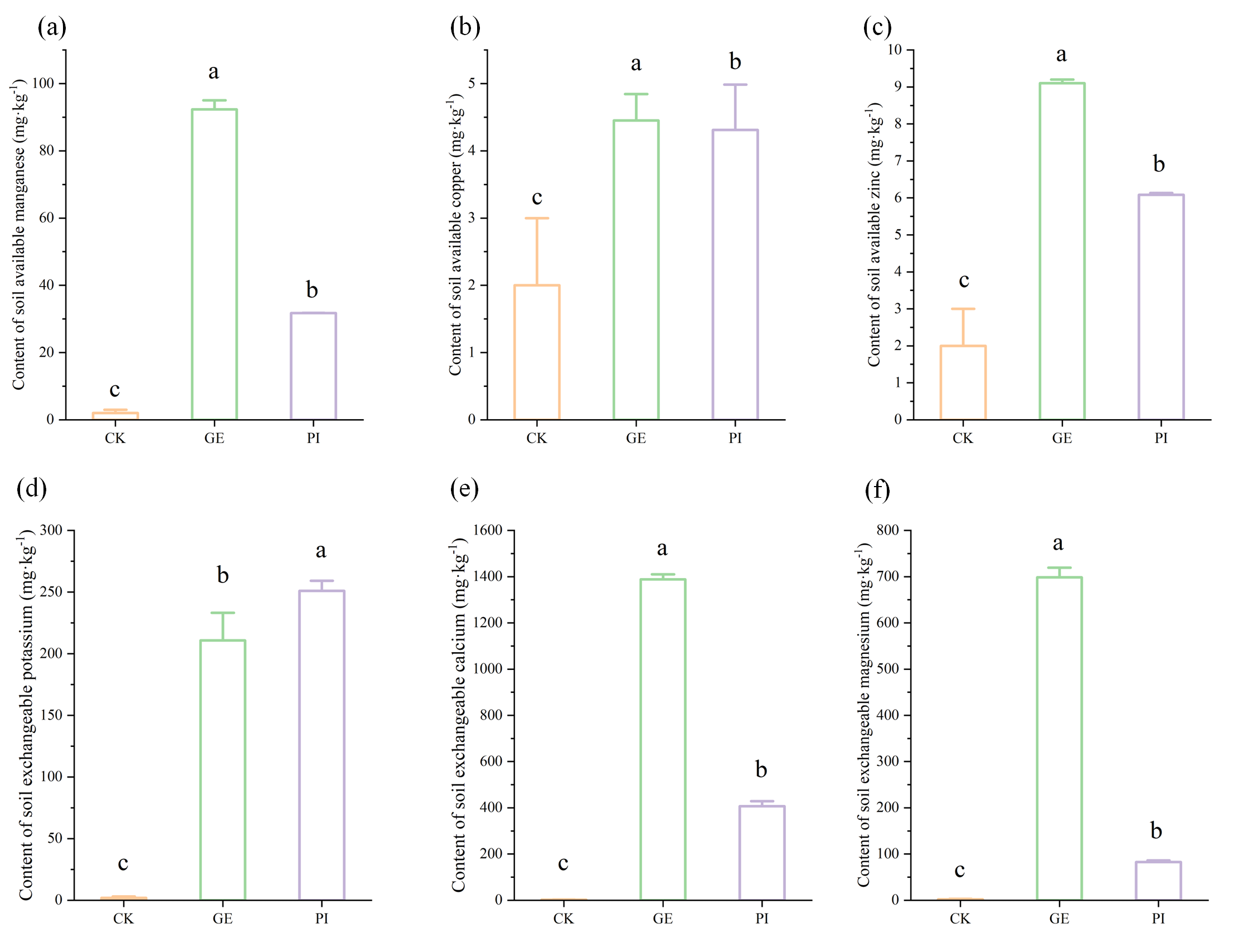
**

**Fig. S2.** Variations of soil available elements and exchangeable cations content under different cultivation management systems. Panels a, b, and c represent soil available manganese, copper, and zinc; panels d, e, and f represent soil exchangeable potassium, calcium, and magnesium. Abbreviation: CK, GE, and PI represent soil samples collected from fallow field (control), soil adhering to the *G. elata*, and soil surrounding the *P. impudicus*, respectively. Lowercases a, b, and c in figures indicate significant differences among treatments (CK, GE and PI), analyzed by one-way ANOVA and Duncan’s multiple range test (*p* < 0.05).

**Table S1**

Humus composition and properties in response to different cultivation management systems

| Treatments | C content in humin | C content in humic acid | C content in fulvic acid | ratio of humic to fulvic acid | humification |
| --- | --- | --- | --- | --- | --- |
|  | g/kg |  |  |  |  |
| CK | 8.65 ± 0.84 b | 3.35 ± 1.27 b | 6.29 ± 1.82 b | 0.51 ± 0.05 b | 0.34 ± 0.02 b |
| GE | 6.52 ± 0.65 b | 0.93 ± 0.02 b | 2.12 ± 0.15 b | 0.44 ± 0.02 b | 0.31 ± 0.01 b |
| PI | 52.9 ± 3.43 a | 12.9 ± 0.76 a | 19.4 ± 1.19 a | 0.67 ± 0.04 a | 0.40 ± 0.01 a |

The different letters within a column represent significant differences among treatments (*p* ≤ 0.05), determined by one-way ANOVA and Duncan’s multiple range test. The data are the means of 3 replicates ± standard error. Abbreviation: CK, GE, and PI represent soil samples collected from fallow field (control), soil adhering to the *G. elata*, and soil surrounding the *P. impudicus*, respectively.
